# Quantifying Rift Valley fever virus transmission efficiency in a lamb-mosquito-lamb model

**DOI:** 10.3389/fcimb.2023.1206089

**Published:** 2023-12-18

**Authors:** Gebbiena M. Bron, Paul J. Wichgers Schreur, Mart C. M. de Jong, Lucien van Keulen, Rianka P. M. Vloet, Constantianus J. M. Koenraadt, Jeroen Kortekaas, Quirine A. ten Bosch

**Affiliations:** ^1^ Quantitative Veterinary Epidemiology, Wageningen University and Research, Wageningen, Netherlands; ^2^ Wageningen Bioveterinary Research, Wageningen University and Research, Lelystad, Netherlands; ^3^ Laboratory of Entomology, Wageningen University and Research, Wageningen, Netherlands

**Keywords:** animal models, arbovirus, epidemiological modelling, biting rate, mosquito-borne disease, Phlebovirus riftense, vector-borne disease, zoonosis

## Abstract

Rift Valley fever virus (RVFV) is a (re)emerging mosquito-borne pathogen impacting human and animal health. How RVFV spreads through a population depends on population-level and individual-level interactions between vector, host and pathogen. Here, we estimated the probability for RVFV to transmit to naive animals by experimentally exposing lambs to a bite of an infectious mosquito, and assessed if and how RVFV infection subsequently developed in the exposed animal. *Aedes aegypti* mosquitoes, previously infected via feeding on a viremic lamb, were used to expose naive lambs to the virus. *Aedes aegypti* colony mosquitoes were used as they are easy to maintain and readily feed in captivity. Other mosquito spp. could be examined with similar methodology. Lambs were exposed to either 1-3 (low exposure) or 7-9 (high exposure) infectious mosquitoes. All lambs in the high exposure group became viremic and showed characteristic signs of Rift Valley fever within 2-4 days post exposure. In contrast, 3 out of 12 lambs in the low exposure group developed viremia and disease, with similar peak-levels of viremia as the high exposure group but with some heterogeneity in the onset of viremia. These results suggest that the likelihood for successful infection of a ruminant host is affected by the number of infectious mosquitoes biting, but also highlights that a single bite of an infectious mosquito can result in disease. The per bite mosquito-to-host transmission efficiency was estimated at 28% (95% confidence interval: 15 - 47%). We subsequently combined this transmission efficiency with estimates for life traits of *Aedes aegypti* or related mosquitoes into a Ross-McDonald mathematical model to illustrate scenarios under which major RVFV outbreaks could occur in naïve populations (i.e., R_0_ >1). The model revealed that relatively high vector-to-host ratios as well as mosquitoes feeding preferably on competent hosts are required for R_0_ to exceed 1. Altogether, this study highlights the importance of experiments that mimic natural exposure to RVFV. The experiments facilitate a better understanding of the natural progression of disease and a direct way to obtain epidemiological parameters for mathematical models.

## 1 Introduction

Rift Valley fever virus (*Phlebovirus riftense*, RVFV) is a mosquito-borne, zoonotic virus within the *Phenuiviridae* family, order of *Bunyavirales*, that mostly affects ruminants (Daubney et al., 1931; Bird et al., 2009). In ruminants, the virus may cause abortion storms and juvenile mortality, with sheep being most affected (Findlay, 1932; Ikegami and Makino, 2011). In humans, Rift Valley fever (RVF) occurs incidentally. The infection is generally self-limiting, but may progress to severe disease with fatal outcome (Wright et al., 2019). Humans become infected by RVFV through contact with infected tissues, e.g., at slaughter, during abortion by contact with placenta and fluids, or after being bitten by infectious mosquitoes (Archer et al., 2013). Over 40 mosquito species, mostly *Aedes* and *Culex* spp., have been found to transmit RVFV under laboratory conditions (Lumley et al., 2017). This may explain the wide geographic range of the virus throughout Africa and highlight a risk for further geographic expansion (Bron et al., 2021). RVF outbreaks have greatly impacted regional economies and the livelihoods of millions of people (Chengula et al., 2013). Due to its potential to cause a public health emergency in absence of efficacious countermeasures, the World Health Organization identified RVFV as a pathogen in urgent need for accelerated research and vaccine development (Mehand et al., 2018). In addition, its demonstrated ability to spread across large geographical areas and cause epizootics and epidemics in new regions calls for a better understanding of RVFV epidemiology.

**Figure 1:**
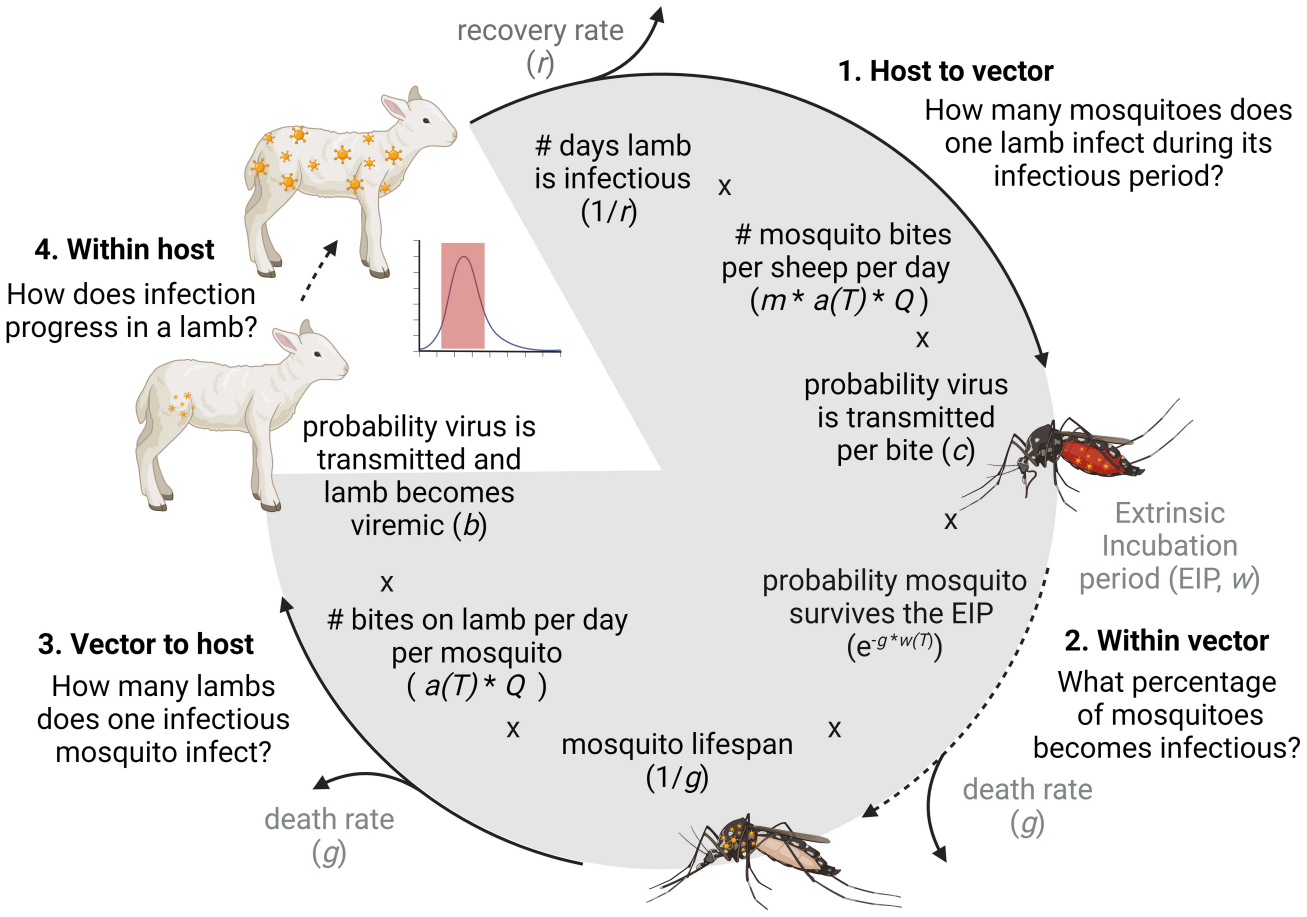
A model of the RVFV transmission cycle and the components of the basic reproduction number (R_0_). The calculation estimates how many new viremic lamb(s) originate from one viremic lamb. In our experiment we estimated *c* (the probability that virus is transmitted from a viremic sheep to a mosquito), *b* (the probability a sheep is successfully infected and becomes viremic after an infectious mosquito bite) and we assessed the within host dynamics of RVFV after low and high mosquito exposure. The other parameters were extracted from literature or were varied to reflect a range of scenarios. *m* = vector to host ratio; *a(T)* temperature dependent biting rate including adjustment for multiple biting behavior (i.e. number of blood meals per mosquito per day); *Q* the proportion of bites on sheep (competent host) versus non-competent hosts, this parameter is affected by host availability and mosquito host preference.

The ability of RVFV strains to cause outbreaks and establish in new places depends on many factors, including the nature and frequency of interactions between vectors and vertebrate hosts. Whether these individual interactions accumulate to cause outbreaks, can be informed by the basic reproductive number (**Figure 1**): the average number of newly infected hosts that arise from a single infected host over the course of its infectious period, in a fully susceptible host population. Only if R_0_ surpasses 1, major outbreaks may occur in naive populations. The quantification of R_0_ relies on the estimation of several epidemiological parameters, some inherent to specific host, vector, or pathogen populations, some highly context specific and dependent on the local environment (**Figure 1**). One specific parameter is the transmission efficiency between vector and host. This metric denotes the probability that an interaction between an infectious mosquito and susceptible hosts results in successful infection. For RVFV, various experimental studies have contributed to an increased understanding of the ability of vertebrate hosts and mosquito vectors to transmit the virus. However, quantification of the efficiency of pathogen transmission can be challenging, particularly in natural host species.

Conventional vector competence and host competence studies may provide an incomplete quantitative understanding of the efficiency of pathogen transmission, due to, for example, the artificial nature of RVFV exposure or the use of indirect transmission metrics (i.e. the detection of virus in mosquito saliva). Positive mosquito saliva demonstrates that virus can pass physiological and immunological barriers in the vector. However it is uncertain, and may underestimate, if and how often successful infection of a host will occur (Gloria-Soria et al., 2022). Experimental animal studies further illustrate that the outcome of infection is not only dependent on the host species and virus strain used, but the outcome is also affected by the mode of RVFV challenge (e.g., needle vs. mosquito bite), the virus cultivation method (e.g., different cell lines or mammalian hosts), and the dose of the inoculum (Smithburn et al., 1949; Turell, 1988; Turell et al., 2008; Le Coupanec et al., 2013; Weingartl et al., 2014; Wichgers Schreur et al., 2021). The development and use of model systems that more closely mimic the natural transmission of RVFV between mosquito vectors and its natural hosts could help resolve such issues.

Host-vector-host models, which investigate transmission of a virus from a mammalian host to a mosquito vector and back, address all parts of an arbovirus transmission cycle as illustrated in **Figure 1**. These studies are particularly powerful because they include many of the natural barriers that pathogens encounter in mammalian hosts and mosquito vectors, as well as possible enhancement of infection by salivary components (Schneider and Higgs, 2008; Guerrero et al., 2020). These mosquito exposure studies are relevant to assess pathogenesis or vaccine efficacy at an individual level. However, a relatively high number of mosquitoes is often used in these mosquito exposure studies to ensure successful pathogen transmission (Dudley et al., 2017; Wichgers Schreur et al., 2021). Such high exposure studies are not suitable for assessing transmission efficiency, as all animals become infected. Furthermore, infection through a single or few mosquito bites is likely more closely mimicking natural exposure, considering relatively low arbovirus vector infection prevalence in the field (McIntosh et al., 1980; Diallo et al., 2005).

The choice of host and vector species for host-mosquito-host models require careful consideration, both on biological meaningfulness and logistical feasibility. The host species used in host-mosquito-host models needs to be suitable for animal experiments, susceptible to the virus in captivity, develop a high enough viremia for the vector to become infected and, ideally, be associated with the natural transmission cycle. With respect to RVF, sheep are most susceptible to infection with short intense viremias often peaking two days post exposure and lasting up to five days (McIntosh and Dickinson, 1973; Weingartl et al., 2014; Faburay et al., 2016; Bron et al., 2021; Wichgers Schreur et al., 2021). Based on experimental studies, lambs developed higher viremia compared to goat kids and calves (Wichgers Schreur et al., 2020), though different breeds of sheep may have different outcomes of infection (Faburay et al., 2016). The mosquito species used in host-mosquito-host models needs to survive the extrinsic incubation period in captivity and be willing to feed in captivity for a second time. Ideally, this would also be a field relevant vector, but these species may not thrive in laboratory colony settings. *Aedes aegypti* colonies are well adapted to feeding in artificial settings. In addition, this species can become infected with RVFV and dependent on the origin/strain of the colony also able to transmit the virus (McIntosh et al., 1980; Turell et al., 2008; Wichgers Schreur et al., 2021). However, *Ae. aegypti* is unlikely to be an important vector in natural settings as, despite its presence in some RVFV endemic regions, it has not been found associated with significant RVFV transmission (Diallo et al., 2005; Seufi and Galal, 2010; Faye et al., 2014; Sow et al., 2014; Sow et al., 2016).

Moreover, laboratory studies on mosquito-borne pathogen transmission help provide a better understanding of the population-level impact pathogens may have. Translating experimental findings to meaningful epidemiological projections requires a thorough understanding of how vectors and hosts interact with each other in different environments, as well as reliable estimates of 1) individual-level outcomes of infection, and 2) the efficiency of transmission from host to vector and vice-versa. To fill some of these knowledge gaps, we experimentally reproduced and quantified parts of the host-vector-host RVFV transmission cycle using *Ae. aegypti* and a European sheep breed with an established record of susceptibility to RVFV (Vloet et al., 2017; Wichgers Schreur et al., 2021). Specifically, we estimate transmission efficiencies from mosquito-to-host and vice versa using biologically relevant experimental exposure models. Next, we describe clinical outcomes and within-host progression of viremia in infected sheep after high and low mosquito exposure. We conclude by using a Ross-McDonald derived model to illustrate how the transmission efficiency estimates relate to invasion risks of RVFV in naive populations, under different scenarios.

## 2 Material and methods

### 2.1 Materials

#### 2.1.1 Cells and viruses

Culture media and supplements were obtained from Gibco unless indicated otherwise. Baby Hamster Kidney (BHK-21) cells were routinely maintained in Glasgow minimum essential medium (GMEM) supplemented with 4% tryptose phosphate broth, 1% minimum essential medium nonessential amino acids (MEM NEAA), 1% antibiotic/antimycotic (a/a) and 5% fetal bovine serum (FBS), at 37°C with 5% CO_2_.

The challenge virus stock of RVFV strain 35/74, a strain originally isolated from the liver of a sheep that died during a RVFV outbreak in the Free State province of South Africa in 1974 (Barnard, 1979), was obtained by low multiplicity of infection (MOI: 0.005) of BHK-21 cells in the presence of CO_2_-independent medium (CIM, Invitrogen), supplemented with 5% FBS (Bodinco) and 1% Pen/Strep (Invitrogen) (Kortekaas et al., 2011).

For assessment of the presence of infectious RVFV in saliva samples, Vero E6 (ATCC, CRL-1586) cells were used. Cells were routinely maintained in Earl’s minimum essential medium (MEM) supplemented with, 1% L-glutamine, 1% MEM NEAA, 1% a/a and 5% FBS, at 37°C with 5% CO2.

#### 2.1.2 Mosquitoes and feeding

Rockefeller strain *Ae. aegypti* mosquitoes (Bayer AG, Monheim, Germany) were routinely maintained at the Laboratory of Entomology of Wageningen University and Research (Wageningen, the Netherlands) as described (Visser et al., 2020). Briefly, mosquitoes were kept in Bugdorm-1 rearing cages at a temperature of 27°C with a 12:12 light:dark cycle and a relative humidity of 70%. The mosquitoes were provided with a 6% sucrose solution ad libitum. Mosquitoes were subsequently transported to biosafety level three (BSL-3) facilities of Wageningen Bioveterinary Research (Lelystad, the Netherlands) for the animal experiment.

#### 2.1.3 Sheep

Texel-Swifter lambs (*Ovis aries*) were obtained from a conventional sheep farm in the Netherlands. Before inclusion in the study, the general health of lambs was assessed by a veterinarian. Animals were allowed to acclimatize in the BSL-3 facilities for 7 days before the start of the experiment. Food and water were available *ad libitum*.

### 2.2 Animal experiment

#### 2.2.1 Study design

**Figure 2:**
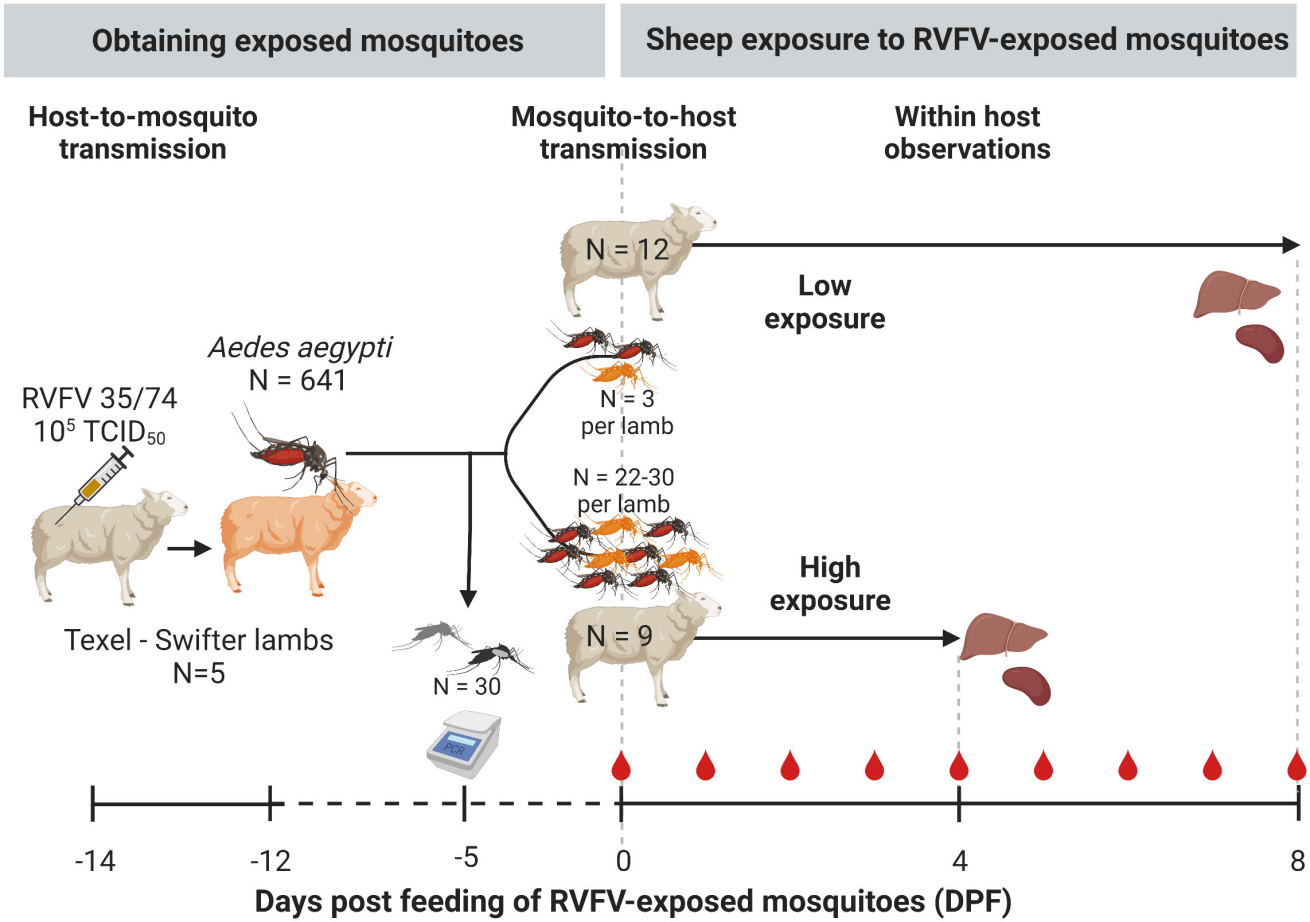
Experimental design. Donor Texel-Swifter lambs were infected by intravenous injection of Rift Valley fever virus (RVFV) two days prior to feeding ~1000 laboratory reared *Aedes aegypti*. All blood fed mosquitoes were subsequently pooled, and kept in a climate and light controlled incubator in a BSL3 laboratory. To estimate RVFV infection rates, 30 mosquitoes were tested for RVFV infection and presence of virus in saliva by RT-qPCR. Twelve days post initial feeding mosquitoes were allowed to take a second blood meal; three (low-exposure group) or 28-31 mosquitoes (high exposure group) were placed in mesh screen containers and allowed to feed on the shaven inner thigh of 10-week-old naive lambs. After mosquito exposure, the lambs were monitored 2-3 times daily for abnormalities and rectal temperatures were measured. Blood samples were taken (red drop in figure). Animals were euthanized and during necropsy liver and spleen samples were collected for detection of RVFV.

A high and low exposure study was conducted using a host-mosquito-host transmission model (**Figure 2**). Following needle inoculation of five lambs with RVFV, mosquitoes *(Aedes aegypti)* were allowed to take a blood meal two days post inoculation, which was previously shown to correspond to peak viremia. Fully engorged mosquitoes were subsequently maintained for 12 days whereafter they were offered a second blood meal this time on naive lambs. Groups of naive lambs were either exposed to a small number of mosquitoes (3; low exposure group) or a larger group of mosquitoes (28-31; high exposure group). Following mosquito feeding, progression of infection was assessed in the lambs of the two exposure groups, health status was monitored and viremia levels were determined daily.

#### 2.2.2 Ethical approval

The animal experiment was conducted in accordance with European regulations (EU directive 2010/63/EU) and the Dutch Law on Animal Experiments (WoD, ID number BWBR0003081). Permissions were granted by the Dutch Central Authority for Scientific Procedures on Animals (Permit Numbers: AVD4010020185564 and AVD4010020187168). All procedures were approved by the Animal Ethics Committees of Wageningen Research. The following humane endpoints were applied: (1) the animal is recumbent and does not rise even after stimulation, (2) the animal is unable to drink, (3) the animal is lethargic (listless, apathic, non-responsive to stimuli).

#### 2.2.3 Obtaining infectious mosquitoes

A group of 10-week-old lambs (N=5) were housed in a BSL-3 animal facility and intravenously inoculated with 10^5^ TCID_50_ of RVFV strain 35/74 at day -14 (**Figure 2**). Two days post infection (day -12) at expected peak viremia, lambs were sedated by intravenous administration of medetomidine (Sedator, Eurovet Animal Health). When fully sedated, cardboard containers with mosquito netting containing 40–50 naive female *Ae. aegypti* mosquitoes were placed on the shaved inner thigh of each hind leg and mosquitoes were allowed to take a blood meal. After approximately 20-30 min of feeding, cardboard containers were removed and animals were euthanized by intravenous injection of an overdose of sodium pentobarbital (Euthasol 20%, AST Pharma). Mosquitoes were transported to a BSL-3 laboratory, where fully engorged mosquitoes were separated from unfed individuals using an automated insect aspirator and pooled in one group. For the next 12 days engorged mosquitoes were maintained in an insect incubator (KBWF 240, Binder) at 28°C, 70% relative humidity and a 16:8 light:dark cycle. Mosquitoes were provided with 6% sucrose solution *ad libitum*. The expected average extrinsic incubation period (*w*) at a 28°C incubation temperature is 10.5 days (**Table 1**) (Turell et al., 1985; Turell, 1989; Barker et al., 2013). However, to account for variability in the EIP between mosquitoes and to ensure that most blood fed mosquitoes have finished their EIP by the time they were allowed to blood feed again, we used a longer waiting time of 12 days (**Figure 2**).

**Table 1:** Description of parameters used in R_0_ calculation.

Parameter		Formula/value used	Reference
Mosquito life history			
Gonotrophic cycle (days)	*gc*	2 + 1/(0.018*T*-0.066)	(Reisen et al., 1992; Barker et al., 2013)
Double feeding proportion	*df*	0.12	(Harrington et al., 2014)
Biting rate (per day)	*a*	(1+*df)*/*gc*	
Deathrate (per day)	*g*	0.09	(Focks et al., 1993)
RVFV specific vector and host traits			
RVFV transmission host to vector (probability)	*c*	27/87 = 0.31	This study
Extrinsic incubation period (days)	*w*	1/(0.0071*T*-0.1038)	(Turell et al., 1985; Turell, 1989; Barker et al., 2013)
RVFV transmission vector to host (probability)	*b*	0.28	This study
Infectious period host (days)	1/*r*	4	This study (Barker et al., 2013; Oymans et al., 2020; Cecilia et al., 2022)
Simulated environment			
Moquito-to-host ratio	*m*	1 - 1500	
Proportion of blood meals from competent hosts	*Q*	0.8	Opportunistic feeding on sheep
		0.2	Anthropophilic feeding or feeding on a non-RVFV competent host
Temperature (˚C)	*T*	28	Incubation temperature in this study

#### 2.2.4 Determining the mosquito exposure protocol for low exposure group

To optimize the chance of exposing lamb in the low exposure group to one mosquito with RVFV in saliva (an infectious mosquito), the following protocol was used: At experimental day –5 a sample group of 30 RVFV-exposed mosquitoes was assessed for virus dissemination in their bodies as described in the section ‘2.3 Sample processing’. The infection prevalence obtained (~1/3) was used to determine the number of mosquitoes allowed to feed per lamb (n=3) in the low exposure group. After feeding on naive lambs on experimental day 0, the infection status of individual mosquitoes was determined. If all mosquitoes would have tested negative for RVFV, a secondary exposure of animals in the low exposure group was planned two days after the initial exposure date.

#### 2.2.5 Exposing naive lambs to RVFV infected mosquitoes

On day 0 of the experiment, nine naive 10-week-old lambs were exposed to 28-31 randomly selected mosquitoes that were fed 12 days prior on needle infected lambs (high exposure group, **Figure 2**). Another 12 lambs were exposed to three mosquitoes each (low exposure group). Exposure of the lambs was performed as described in the section ‘2.2.3 Obtaining infectious mosquitoes’.

For the low exposure group, feeding of three mosquitoes was monitored in real-time and when less than three mosquitoes had fed (from the initial container with three mosquitoes) additional single mosquitoes were placed on a lamb until three mosquitoes had fed on each animal. To estimate bites per animal for the high exposure group, engorged mosquitoes were separated from unfed mosquitoes and counted within the first day post feeding. Following separation and counting, a subset of six mosquitoes per lamb (high exposure group) was randomly selected and subjected to forced salivation and assessment of virus in their bodies. All fed mosquitoes of the low exposure group were tested similarly.

#### 2.2.6 Monitoring of mosquito-exposed lambs

Following mosquito exposure, lambs were monitored three times daily for clinical signs and rectal temperatures were measured twice daily. Blood samples were taken on a daily basis to assess viremia (**Figure 2**). The high exposed lambs were euthanized and necropsied 4 DPF to prevent unnecessary discomfort, anticipating that high exposure would result in severe disease. The low exposed animals were euthanized at 8 DPF. At necropsy, spleen and liver samples were collected.

### 2.3 Sample processing

#### 2.3.1 Lambs

To assess RVFV infection in lambs, RNA was isolated with the NucliSENS easyMAG system according to the manufacturer’s instructions (bioMerieux, France) from 0.5 ml plasma samples. To detect RVFV genetic material RT-qPCR was conducted. Briefly, 5 µl RNA eluate was used in a RVFV RT-qPCR using the LightCycler one-tube RNA Amplification Kit HybProbe (Roche, Almere, The Netherlands) in combination with a LightCycler 480 real-time PCR system (Roche) and the RVS forward primers (AAAGGAACAATGGACTCTGGTCA), the RVAs (CACTTCTTACTACCATGTCCTCCAAT) reverse primer and a FAM-labelled probe RVP (AAAGCTTTGATATCTCTCAGTGCCCCAA). Primers and probes were earlier described by Drosten et al. (2002). Virus isolations were performed on RT-qPCR positive samples with a threshold above 10^5^ RNA copies/ml as this was previously shown to be a cut-off point below which no live virus can be isolated.

#### 2.3.2 Mosquitoes

To determine if mosquitoes were infected with RVFV, viral RNA was isolated from homogenized mosquitoes and from saliva samples. Briefly, bodies were homogenized in 300 μl Trizol with a pellet pestle (Sigma) and the homogenate was subsequently cleared by slow speed centrifugation. Individual saliva samples (see 2.3.3 Virus isolation) were also added to 300 μl Trizol. Total RNA was subsequently isolated using the Direct-zol™ RNA MiniPrep kit (Zymo Research) according to the manufacturer’s instructions. The level of RVFV RNA was subsequently determined as described in section ‘2.3.1 Lambs’.

#### 2.3.3 Virus isolation

To determine the presence of infectious RVFV in mosquito saliva, plasma and tissue samples from lambs, virus isolations were performed. To check for positive saliva, mosquitoes were sedated on a semi-permeable CO_2_-pad connected to 100% CO_2_ and wings and legs were removed. Saliva was collected by forced salivation using 20 µl filter tips containing 7 µl of a 1:1 mixture of FBS and 50% sucrose (capillary tube method). After 1–1.5 h, saliva samples were collected and incubated with Vero-E6 cell monolayers. Cytopathic effect (CPE) was scored 5–7 days later. To detect RVFV in plasma, serial dilutions of plasma samples were incubated with 20,000 BHK-21 cells/well in 96-wells plates for 1.5 h before medium replacement. Cytopathic effect was evaluated after 5–7 days post infection. Virus titers (TCID_50_/ml) were determined using the Spearman-Kärber algorithm. The limit of detection was 1.55 log_10_ TCID_50_/ml.

#### 2.3.4 Serology

Antibodies against RVFV nucleoprotein (RVFV-NP) were detected by semi-quantitative ID Screen^®^ Rift Valley Fever Competition Multi-species (IDVet, Grabels, France) enzyme-linked immunosorbent assay (ELISA) on a weekly basis. The ELISA was conducted according to the manufacturer’s instructions. Briefly, RVFV-NP was coated in wells, anti-RVFV-NP antibodies in sera were allowed to bind, and anti-NP-peroxidase was used to bind to free RVFV-NP in the well (i.e., RVFV-NP not blocked by anti-NP antibodies). A wash protocol occurred between each step. Finally, substrate (TMB) for bound peroxidase was added, and plates were read out at 450nm. Titers are expressed as percentage inhibition ratio of the optical densities (OD) of the sample and the OD of the negative control (S/N * 100). All values lower than 40% are considered positive, between 40 and 50% are considered doubtful and above 50% are considered negative.

### 2.4 Data analyses

#### 2.4.1 Descriptive statistics

Demographics (age, sex), health status (rectal temperature, survival), and viral infection characteristics (presence of viremia [yes or no], day of onset of viremia, day of peak viremia, and peak viremia level) were summarized. Rectal temperatures (T_r_) from DPF 1 to 4 (last day of study period for the high exposure group) were included to calculate the mean T_r_ and standard deviation per group. A temperature of 40.5˚C or higher was considered a fever. To determine the day of fever onset, morning observations were coded as DPF and afternoon observations as DPF + half a day. An animal was considered viremic when the TCID_50_ was above the limit of detection. The probability of developing viremia and mortality were compared between the two exposure groups using Fisher Exact tests (FET). Peak viremia in log_10_ TCID_50_/ml, day of onset of viremia, day of peak viremia and day of onset of fever were compared between high and low exposure groups by Wilcoxon Rank Sum test. The Pearson’s correlation coefficient of morning T_r_ and log_10_ TCID_50_/ml was calculated. The duration of viremia and fever were not compared between groups, because animals in the high exposure group were euthanized on 4 DPF, to minimize animal suffering, when viremia and fever were still present.

#### 2.4.2 Examining the relationship between mosquito bites and infection outcomes

To examine if and how the probability of acquiring infection, *P*(inf), was affected by the number of infected mosquito bites received by a single host (*n*, the number of mosquitoes that took a blood meal) in a short time frame, we fitted two probabilistic models to the experimental data, each representing a hypothesis of the nature of this possible dose-response relationship.

The models represent two distinct hypotheses: *i*) *P*(inf) is independent of *n*:


$$P\left(Inf\right)=d$$

*ii) P*(inf) is dependent of *n*, where each subsequent infectious bite accrues the same per bite probability of infection (*b*), irrespective of earlier events:


$$P\left(Inf|n\right)=1-{\left(1-b\right)}^{n}.$$

The first hypothesis can be regarded as the null hypothesis and denotes that the probability of infection is stable and not affected by the number of bites. This probability of infection after exposure to infectious mosquito bites (*n*>0) is here denoted by *d*, which can take any value between 0 and 1. This hypothesis may be true especially for highly contagious pathogens where the received pathogen dose is not a limiting factor for transmission success. The second hypothesis reflects what is commonly adopted in mathematical models of mosquito-borne pathogens (Reiner et al., 2013). Herein, the probability for a single bite by an infectious mosquito to cause infection in a host, the vector-to-host transmission efficiency (*b*), is estimated. It assumes that, within an individual sheep, the probability of acquiring infection follows a Poisson process. Models *i* and *ii* were fitted to the experimental data by Maximum Likelihood Estimation and compared using AIC-scores (Burnham and Anderson, 2004).

The number of infected mosquitoes a lamb was exposed to (*n*) was derived differently between exposure groups. In the low exposure group, all mosquitoes were tested for infection and thus provide a direct measure of *n* (PCR positive bodies were used for the reported *b* calculation, because saliva results were not available for all mosquitoes). In the high exposure group, the number of infectious bites was estimated indirectly from a random sample of six mosquitoes per lamb. The number of engorged mosquitoes per individual in the high exposure group was multiplied with the proportion saliva positive mosquitoes to estimate the number of infectious bites (*n*) per lamb. A sensitivity analysis was performed to assess the impact of uncertainty in the estimate of the number of infectious mosquitoes (**Supplementary Table 1**).

#### 2.4.3 Illustrating epidemiological implications

To explore if and under which circumstances RVFV transmission by mosquitoes with similar life history and transmission traits as *Ae. aegypti* could result in RVFV outbreaks, we applied the experimental estimates on the probability of transmission from mosquito to sheep (*b*) and sheep to mosquito (*c*) in a Ross-MacDonald-like framework for the basic reproduction number (Smith et al., 2007). The basic reproduction number here is defined as the average number of new infected sheep that are expected to arise from a single infected sheep over the course of its infectious period, in a fully susceptible population. It is a commonly used metric of transmission potential, as only if R_0_ surpasses 1, major outbreaks may occur. It follows


$$R_{0}=\frac{m{(Qa(T))}^{2}bce^{-gw(T)}}{rg}.$$

This definition follows from the several ‘hurdles’ a mosquito-borne pathogen needs to overcome to fulfil its transmission cycle (**Figure 1**). Parameter definitions and default values are depicted in **Table 1**. Here, the length of the extrinsic incubation period (*w*) and biting rate (*a*) are considered to change as a function of temperature. The biting rate is the inverse of the gonotrophic cycle (*gc*) length under the assumption that mosquitoes take a single blood meal per *gc*. Departure from this assumption is allowed for by including double feeding behavior $\left(1+df\right)/gc$ , where *df* denotes the proportion of mosquitoes that take a second blood meal during their *gc*. Mosquitoes are expected to remain infected for the remainder of their lives. Bites are assumed to be homogeneously distributed (i.e., every competent host has the same probability of getting bitten. The vector-to-host ratio (*m*) and the proportion of blood meals to be taken from a competent host (*Q*) are considered context specific, for example high vector-to-host ratios could be expected during rainy seasons, and bites on competent host would be high in farm settings where competent hosts are relatively abundant versus a city. We therefore explored the value of R_0_ in different host biting scenarios and with different vector-to-host ratios, while assuming a temperature equal to our experimental set-up (28°C). For illustrative purposes, we present two ‘extreme’ biting scenarios: vector opportunistically feeding on competent hosts (*Q* = 0.8) or on a preferred non-competent host (*Q* = 0.2). In this illustrative example, we assume all competent hosts to have transmission parameters similar to that of sheep. We illustrated the sensitivity of R_0_ to *b* by considering values of *b* from 0.05 to 0.95. Parameters were taken from literature specific to RVFV or *Ae. aegypti*, with the exception of the duration of the extrinsic incubation period by temperature. By lack of an *Ae. aegypti* specific estimate, the formula estimated by Barker et al. (2013) based on experiments by Turell et al, 1985; Turell, 1989 using *Aedes fowleri* (17 and 28°C) and *Ae. taeniorhynchus* (26 and 33°C) were used.

### 2.5 Software

All animal observations were logged in an iVention notebook. Analyses were conducted with R Statistical Computing Software: *bbmle* (version: 1.0.24) was used for maximum likelihood estimation and AIC comparison, *binom* (version 1.1-1) for binomial confidence estimates calculation, and *ggplot2* (version 3.3.5) for visualizing data and model results (Dorai-Raj, 2014; Wickham, 2016; R Core Team, 2020; Bolker and Development Core Team, 2021).

## 3 Results

### 3.1 Host-to-mosquito transmission: obtaining RVFV exposed mosquitoes

To obtain infectious mosquitoes, approximately 1,000 female naive mosquitoes were allowed to take a blood meal from lambs that were needle inoculated with 10^5^ TCID_50_ RVFV two days prior. All five donor lambs had detectable fever at the time of mosquito feeding. A total of 641 mosquitoes fed on the lambs, these mosquitoes were pooled and maintained in an incubator until further use. Retrospectively, four out of five lambs had developed high viremia; one animal was viral RNA positive, but had an RVFV titer at or below the limit of detection (**Supplementary Table 2**). The average RVFV titer in the blood of the lambs was 6.59 log_10_ TCID_50_/ml (range: limit of detection 1.55, 7.15).

To estimate the proportion of mosquitoes that acquired infection (RVFV-positive bodies) and became infectious (RVFV-positive saliva), a subset of 30 mosquitoes was randomly selected for RT-qPCR testing on day 7 post initial feeding (experimental day -5). Due to logistic reasons, testing at a later time-point was not possible. Viral RNA was detected in 9 out of the 30 bodies tested, suggesting a 30% infection ratio (95% confidence interval [95%CI]: 15 - 49%, **Supplementary Table 3**). Virus was not detected in corresponding saliva samples (0/30). Based on previous experiments, we assumed that following another 5 days incubation, 12 days post initial feeding (experimental day 0), virus would have reached the salivary glands in infected mosquitoes (Wichgers Schreur et al., 2021; Bermúdez-Méndez et al., 2022).

### 3.2 Mosquito-to-host transmission

#### 3.2.1 Exposure of naive lambs to a high or low number of infected mosquitoes

**Figure 3:**
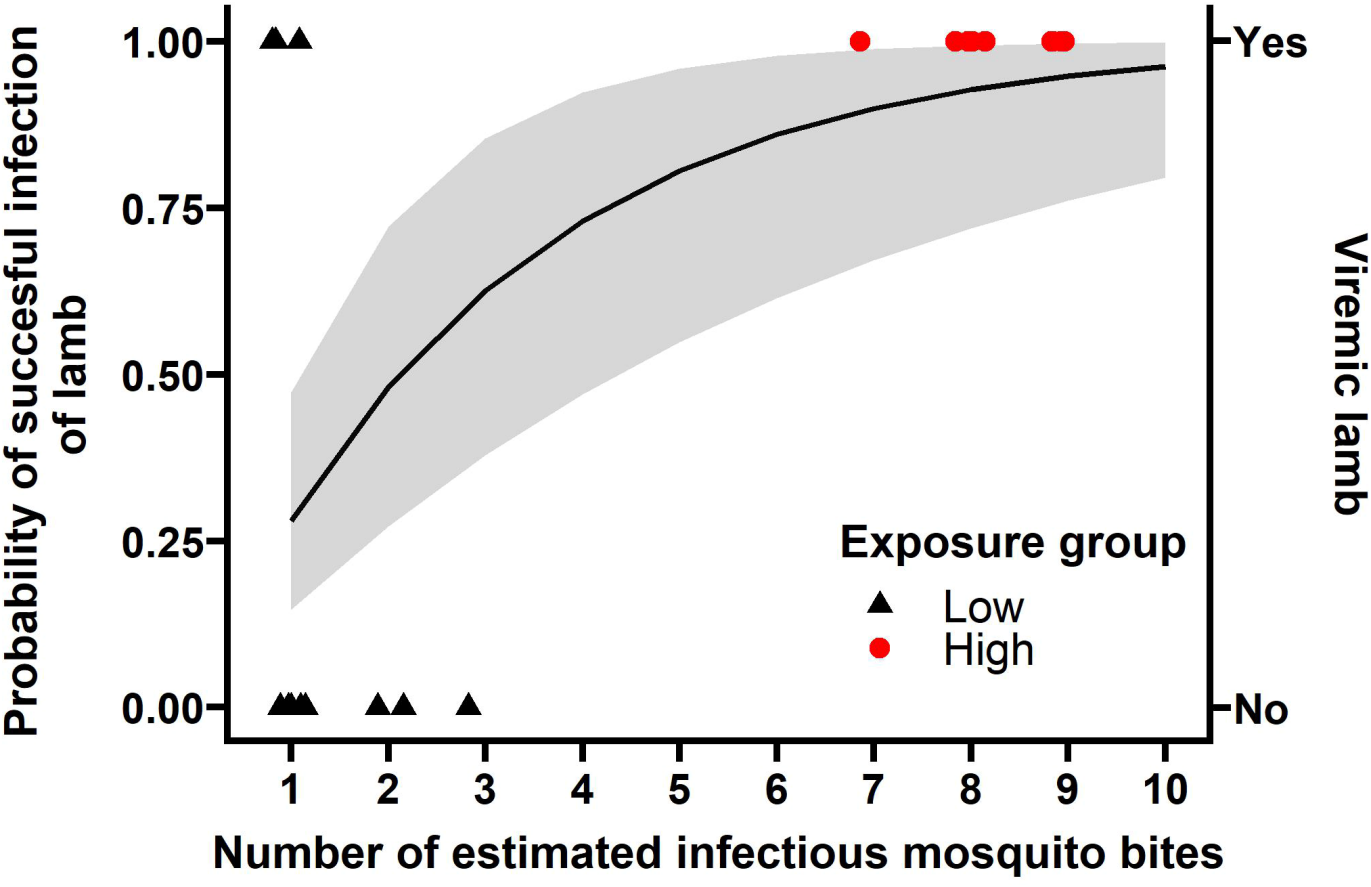
The number of bites matters for successful infection. Shapes represent the data collected (**Supplementary Table 4**), 9 individuals in the high exposure group (red) and 12 in the low exposure group (black) became viremic or not (y-axis on the right). The *b* estimate from the ‘bites matter’ model 0.280 (95%CI: 0.147, 0.473) was used to calculate the probability of infection per number of mosquito bites (black line, and grey ribbon represents its 95% confidence interval, y-axis on the left). Bites in the low exposure group represent PCR positive bodies, bites in the high exposure group represent the number of engorged mosquitoes expected to have RVFV in saliva.

Based on the expected 30% infection ratio of the pool of RVFV-exposed mosquitoes and the aim to expose lambs of the low-exposure group to one bite containing RVFV on average, three mosquitoes were placed on each lamb. A retrospective analysis of these mosquitoes revealed that 9 out of 12 lambs were indeed exposed to a single bite by a mosquito with an RVFV-positive body, whereas two received two and one received three bites (**Figure 3**, **Supplementary Table 4**). Of note, of the nine lambs exposed to a single infected mosquito, five mosquitoes had detectable viral particles in their saliva, three did not and for one mosquito this information was not available.

In the high exposure group, lambs were exposed to 28-31 RVFV-exposed mosquitoes and 22 to 30 mosquitoes were engorged (**Table 2** and **Figure 2**). To estimate the number of infectious bites, saliva was collected from 33 of the blood fed mosquitoes from the low exposure group and a subset of the high exposure group (N=6 per lamb) immediately after feeding for a second time. Saliva samples from 27 of 87 mosquitoes were virus isolation positive (31%, 95% CI: 22 - 42%). Taking the number of engorged mosquitoes and the proportion of RVFV positive saliva samples into account, lambs of the high exposure group were estimated to be exposed to 7-9 infectious bites (**Table 2**).

**Table 2:** Summary of lamb characteristics, challenge and RVFV infection outcomes of high and low mosquito exposure groups. Mean ± SD are shown when applicable.

		High exposure	Low exposure
Study demographics			
Number of sheep		9	12
Sex		6F, 3M	11F, 1M
Age at challenge		10 weeks	10 weeks
Weight		19.4 ± 2.4 kg (range: 13.9, 21.9)	17.6 ± 2.4 kg (range: 13.7, 22.3)
Euthanized		4 DPF	8 DPF
Challenge			
Engorged mosquitoes		27 (range: 22-30)	3
% infectious mosquitoes		31.5% (17/54)	30.3% (10/33*)
Infection outcomes			
Rectal temperature (DPF 1-4)		41.0 ± 0.79°C (range: 39.2, 42.1)	39.9 ± 0.49°C (range: 39.0, 42.0)
Fever	Present	100% (9/9)	42% (5/12)
	Onset	2.2 ± 0.5 DPF (range: 2, 3.5)	3.7 ± 1.5 DPF (range: 2.5, 6)
Animals with viremia		100% (9/9)	25% (3/12)
Viremia onset		2.1 ± 0.3 DPF (range: 2, 3)	3.7 ± 2.1 DPF (range: 2, 6)
Peak viremia	Day	2.8 ± 0.6 DPF (range: 2, 4)	4.0 ± 1.7 DPF (range: 3, 6)
	Titer	6.7 ± 0.5 log10/ml (range: 5.28, 6.91)	6.8 ± 0.4 log10/ml (range: 6.45, 7.15)
Mortality		11% (1/9, 3 DPF)	8% (1/12, 4 DPF)

*The presence of RVFV in saliva was not determined for three mosquitoes, saliva collection was unsuccessful.

DPF, Days post feeding of RVFV-exposed mosquitoes; F, female; M, male.

#### 3.2.2 Clinical outcomes

##### 3.2.2.1 High exposure group

**Figure 4:**
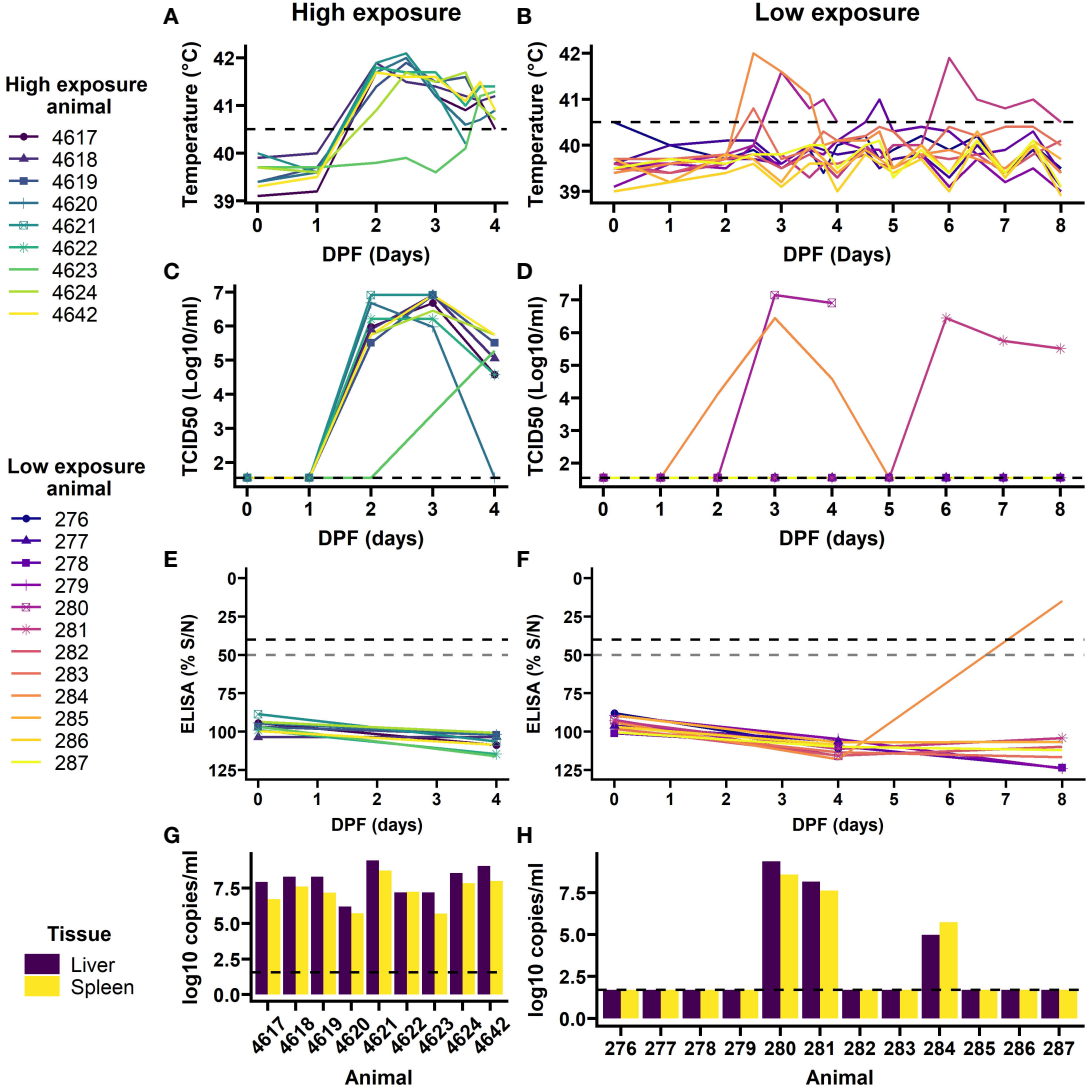
Outcomes of exposure to RVFV-exposed mosquitoes in individual lamb in high exposure (left) and low exposure (right) groups. Rectal temperatures were taken twice daily, and more frequent during 3 and 4 DPF **(A, B)**. Temperatures over 40.5 C were considered a fever (dashed line). Blood samples were daily assessed for viremia development **(C, D)**, limit of detection is shown with the dashed line. At day 0, 4 and 8 DPF anti-RVFV antibody development was assessed by ELISA **(E, F)**. A 50% signal was probable positive and 40% or less was considered positive (dashed lines). Upon euthanasia, RVFV presence was determined in 10% spleen and liver homogenates **(G, H)**. PCR limit of detection shown in dashed line. Animal 4621 and 280 succumbed to infection on 3 and 4 DPF, respectively.

Following mosquito challenge, all (9) high exposure animals presented with increased rectal temperatures from day 2 or 3 onwards (**Figure 4A**). Furthermore, these animals presented with reduced feed intake and became lethargic. One animal succumbed to the infection and the other animals were euthanized at 4 DPF as planned. PCR on plasma samples, followed by virus isolation demonstrated that all nine animals had developed viremia with eight animals presenting with a characteristic RVFV viremia curve starting at 2 DPF and one animal with a one day delay (**Figure 4C**). In addition, viral RNA (**Figure 4G**) and infectious virus were detected in all liver and spleen samples at the time of necropsy, except for the liver sample of animal 4620 in which no infectious virus was detected.

##### 3.2.2.2 Low exposure group

In the low exposure group 9 out of 12 lambs did not present with clinical signs of disease, RVFV specific antibodies, nor presented with viremia and/or virus in liver and spleen samples at the time of necropsy, 8 DPF (**Figures 4B, D, F, H**). However, two animals (#280 and #284) did present with characteristic features of RVFV infection starting at 2 DPF, which included fever, reduced feed intake and lethargy. Animal 280 succumbed to the acute infection at 4 DPF. A third animal (#281) presented with signs of disease at 5 DPF. In line with the clinical signs all three animals presented with high viremia within one or two days post onset of symptoms. Furthermore, liver and spleen samples at the time of necropsy were highly positive for animal 280 and 281. Animal 284 had developed antibodies against RVFV and had cleared the virus at 8 DPF (**Figures 4F, H**).

##### 3.2.2.3 Comparing natural infection between high and low exposure groups

After successful exposure to RVFV through mosquito bite(s) all animals (9/9) in the high exposure group developed viremia compared to 25% (3/12) in the low exposure group (FET *p*<0.001, **Table 2** and **Figure 4**). No difference in mortality ratio was noted; one animal succumbed to RVF in each group (animal 4621 and 280, FET *p*=1, **Table 2**). All animals that developed viremia also presented with a fever (correlation coefficient 0.87, 95%CI: 0.82 - 0.90, t(146)=21.8, p<0.001). In the low exposure group, two additional animals had an afternoon or evening with fever 2 DPF and 4 DPF, but they did not have detectable viral RNA or virus in their blood. No difference in peak viremia level was detected between the groups, peak viremias were 6.91 in the high exposure group and 7.15 log_10_ TCID_50_/ml in the low exposure group (t(2.31)=0.36, *p*=0.75). In the low exposure group, the peaks appeared more variable, but no significant difference was detected in peak day between the two groups (W=21.5, *p*=0.07). None of the sheep had detectable anti-RVFV antibodies at the start of the study (**Figures 4E, F**). The animals in the high exposure group were euthanized before antibodies could develop. No comparison was made with the low exposure group, where one previously viremic animal had detectable antibodies 8 DPF.

#### 3.2.3 Mosquito-to-host transmission efficiency (*b)*

Using the clinical outcomes and the individual-level information on number of infectious bites (**Figure 3** and **Supplementary Table 4**), we estimated the mosquito-to-host transmission efficiency (*b*). We tested whether the probability of acquiring infection is affected by the number of bites received, distinguishing two hypotheses: *i*) no impact of the number of bites on the acquisition of infection (the null hypothesis) or *ii*) the number of bites matters and each additional bite independently increases the infection probability. The model assuming bites to be independent of each other was best supported by the data (Model *ii*, AIC 19.4, delta AIC 11.3).

With model *ii*, we assume the infection probability to follow from a Poisson process in which each infectious bite is considered an independent event. Each independent attempt is estimated to result in infection with a probability (*b*) 0.280 (95%CI: 0.147, 0.473) (**Figure 3**). Using this model, we estimated that a host being exposed to two infectious bites has a near 50% chance of acquiring an infection (48.1%, 95%CI: 27.2, 72.2%).

For the reported estimate of *b* we used the number of body positive mosquitoes when referring to infectious mosquito bites in the low exposure group. For the high exposure group, we used a conventional approach: proportion of saliva positive mosquitoes of those that were engorged. The number of mosquitoes with RVFV positive bodies was used in the low exposure group in order to deal with the missing data points for RVFV in saliva and the observation that one of the sheep exposed to mosquitoes without detectable RVFV in saliva became viremic (animal 281). Suggesting RVFV may have been present in saliva below the limit of detection. Both model selection and estimates for *b* were robust for the use of different definitions of infections (TCID_50_ and PCR) and feeding status (exposed and engorged mosquitoes, **Supplementary Table 1**). Estimates for *b* ranged from 0.216 to 0.319 using different definitions of infectious bites (see **Supplementary Table 1**).

### 3.3 Illustration of epidemiological implications

**Figure 5:**
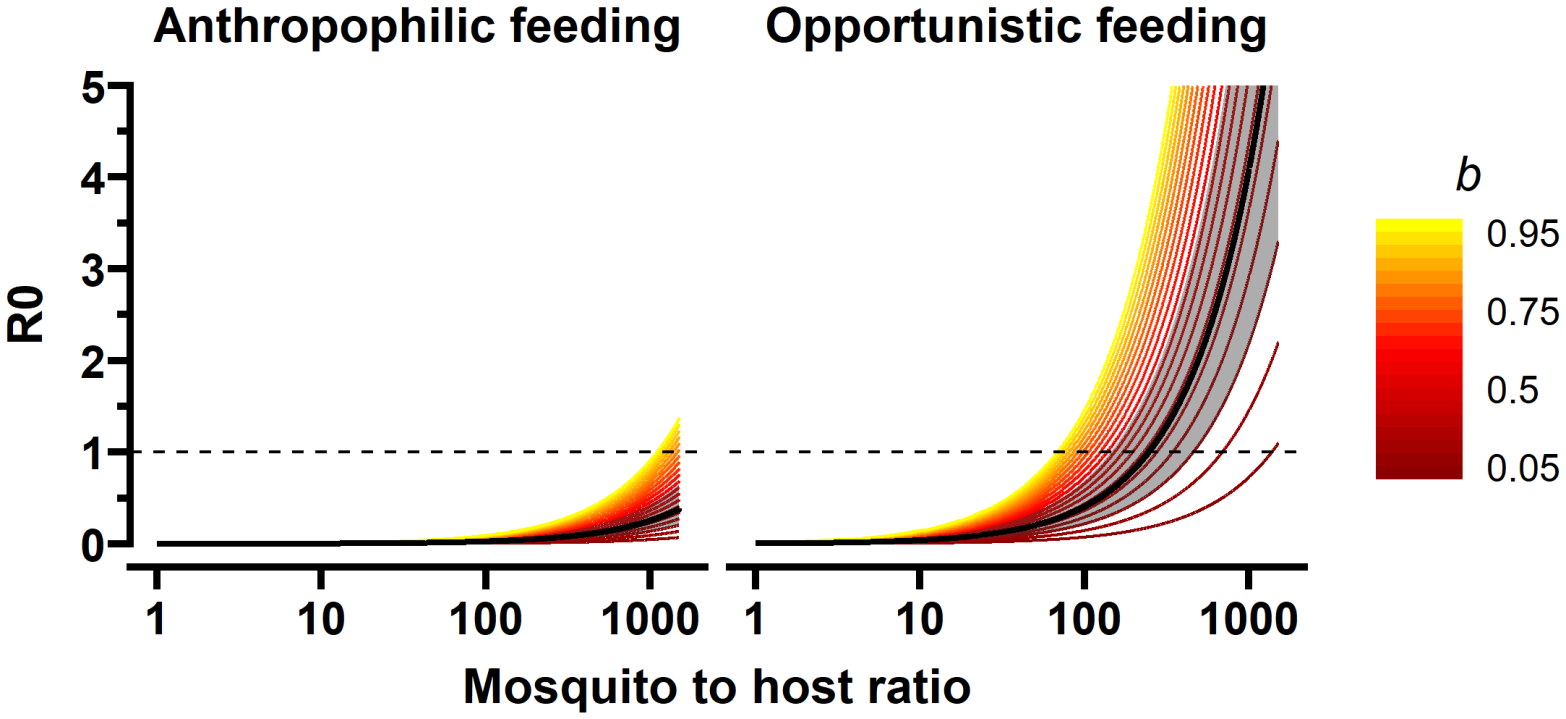
R_0_ for mosquito-to-host transmission efficiencies (*b*) in different contexts. The contexts represent: a mosquito to host ratio (*m*) from 1 to 1500 mosquitoes per lamb, and anthropophilic and opportunistic mosquito feeding behavior (*Q* = 20% versus 80% of blood meals from sheep). The bold black line represents the estimate derived from our experiment (**Figure 2**) and the grey shaded area includes the 95% confidence interval. The colored lines represent *b* from 0.05 to 0.95 from dark red to yellow. The vertical line represents the endemic equilibrium (R_0 _= 1), values above this line indicate that an RVFV outbreak would occur in a naive population under the modeled scenarios, whereby temperature was set at 28°C leading to an extrinsic incubation period (*w*) of 10.5 days and biting rate (*a*) of 0.26 blood meals per mosquito per day, mosquito death rate was 0.09 (an average lifespan of 11 days), the infectious period of lamb was four days and the host-to-vector transmission efficiency 0.31 (*c*).

Given the estimated mosquito-to-host transmission efficiency (*b*) based on our experimental study we subsequently modelled under which circumstances outbreaks with *Ae. aegypti* involvement or alternative vectors with similar transmission and life history traits could occur. Specifically, we calculated the reproduction numbers (R_0_) for different mosquito-to-host densities and two host-feeding scenarios: opportunistic feeding on competent hosts (sheep), or anthropophilic feeding behavior, i.e. 80% of mosquitoes feeding on preferred human host (Ponlawat and Harrington, 2005), which are considered dead-end hosts for RVFV. We show that, for *Ae. aegypti* to be able to cause an outbreak in sheep, it needs to portray opportunistic biting behavior, i.e., in settings where sheep density is high compared to humans, it would need to feed proportionally more on sheep (or other competent ruminant species), despite its preference for feeding on humans (**Figure 5**). In such a scenario, high numbers of mosquitoes (mosquito-to-host ratio, *m >*200) are needed, even at 28°C, to surpass R_0_ = 1. If 80% of bites were on humans (or other dead-end host for RVFV), higher numbers of *Ae. aegypti* would be needed for R_0_ to reach the critical value of unity (*m >*1000, **Figure 5**).

## 4 Discussion

Here, we performed a host-mosquito-host transmission experiment with RVFV in a controlled setting in which lambs were exposed to different numbers of infectious mosquitoes. We estimated the vector-to-host transmission efficiency of RVFV between Texel-Swifter lambs and *Ae. aegypti* mosquitoes and showed that the number of mosquito bites is a major factor in determining whether an animal develops a productive RVFV infection; all animals fed on by 7+ infectious mosquitoes became infected, developed viremia and fever, whereas only a quarter of animals exposed to a single infectious mosquito on average became productively infected. The transmission efficiency from vector-to-host was estimated to be 28% (95% CI: 15 - 47%) and from host-to-vector 31% at peak viremia (95% CI: 22 – 42%). This study marks a unique empirical estimate of transmission efficiency from mosquitoes to a natural host species by a single mosquito bite, an important component of the transmission cycle of RVFV.

RVFV vector-to-host transmission efficiencies as determined under experimental conditions (*b*) can vary depending on the species and biotypes of mosquitoes, virus strain, routes of mosquito infection (e.g., membrane feeding, injection skipping the midgut-barrier, feeding on non-natural host species), and host species used (Turell and Bailey, 1987; Turell et al., 2008). Here we used the proportion of Texel-Swifter lambs that became viremic as our primary outcome measure for successful transmission. With three out of twelve sheep infected after exposure to a single bite by RVFV positive mosquitoes, the transmission of RVFV from *Ae. aegypti* in this study was higher than that of other studies using *Ae. aegypti* as a vector. For instance, one out of seven hamsters (14%) succumbed to RVFV after a bite from *Ae. aegypti* with disseminated infection (Turell et al., 2008). The transmission efficiency is likely reflected by this proportion, as hamsters experience 100% mortality to infection. Further, in early host-vector-host transmission experiments, no transmission was observed following exposure of mice and lamb to eight exposed *Ae. aegypti* mosquitoes (five of which contained RVFV Smithburn et al., 1949). It was however not reported if individual or small groups of mosquitoes were used for animal exposure. Such intricacies make the estimate of mosquito-to-host transmission efficiency (*b* in **Figure 1**) difficult to compare to other studies. For example, 55, 84 and 100% transmission of RVFV was observed from African *Ae. palpalis*, *Culex antennatus* and *Cx.pipiens* with disseminated infection to hamsters, but this could have been based on one to up to five mosquitoes per animal (Turell et al., 2008). Furthermore, care should also be taken in extrapolating vector-to-host probability of transmission by a single bite (*b*) to other ruminant species or other sheep breeds, for instance those present in RVFV endemic countries. Estimates for other species are expected to be lower as sheep are deemed most susceptible to RVFV infection. In addition, we did not consider probing (i.e., unsuccessful blood feeding attempts) as an exposure event, even though these could potentially also contribute to transmission (Matsuoka et al., 2002; Styer et al., 2007). We also did not consider body positive mosquitoes for our denominator in the high exposure group. Instead, we used saliva positive mosquitoes. This may result in an overestimation of mosquito-to-host virus transmission (*b*), because too few mosquitoes are considered capable of transmitting virus (*n*). Furthermore, the method of detecting virus in saliva has imperfect sensitivity (Gloria-Soria et al., 2022). This was also demonstrated by the animal in the low exposure group that did become viremic after exposure to mosquitoes without detectable viral particles in saliva. Using more liberal infection rates for mosquitoes, i.e., using the number of mosquitoes with PCR positive bodies, results in somewhat lower levels of *b* (24%, 95%CI: 12-44%, **Supplementary Table 1**).

This study addresses two topics of arbovirus transmission at the level of the individual host: i) the number of (infectious) mosquito bites individual animals receive determine the probability of acquiring infection, ii) within hosts, if RVFV infection is successful and viremia develops, viremia peak levels appear similar between low and high exposed individuals. Development of viremia and clinical signs in the high exposure group were similar to Wichgers Schreur et al. (2021); rectal temperatures and RVFV titers were strongly correlated and both peaked on 2-3 DPF. This relative short and intense viremia is seen in several other arboviral infections (Binn et al., 1967; Oesterle et al., 2009). This phenomena is often interpreted in light of the virus’ tradeoff between magnitude and duration of viremia. This tradeoff has been observed in experiments with varying dosages, where high dosages result in high-peak, short-lasting viremia, and low dosages result in longer-lasting viremia that peaks at lower levels. The mechanism behind this dose response relationship is not completely clear and may well vary between pathogens, and the interactions with hosts. One explanation is that high initial viremia accelerates both initial virus replication and the recruitment of immune cells. While observed in many arboviruses, the evidence for such a relationship in arboviruses is not consistent (Althouse and Hanley, 2015). One counter example, for instance, follows from West Nile virus studies, which were not able to detect differences in peak viral titers between high and low dose groups after needle inoculation (Oesterle et al., 2009; VanDalen et al., 2013). Similar to these observations in West Nile virus viral kinetics, we found no indication that peak titers were affected by the level of mosquito exposure. However, due to the natural exposure route used in our study we were not able to control exposure dose. Variation in exposure dose could have blurred part of the dose response relationships, if present, particularly in the low exposure group, in which only three animals developed viremia.

Two interesting observations in the low exposure group could be examined further in future studies: the seemingly later onset of viremia, as well as the slow decline in viremia in single animals in this group. The later onset of viremia on 6 DPF in animal 281 may be caused by initial local (skin and or lymph node) replication, before the virus reached the liver and initiated full viremia. This animal was exposed to one RVFV PCR positive mosquito, but the mosquito had no detectable virus in her saliva. Therefore, the RVFV exposure dose for animal 281 was likely very low. An alternative explanation would be direct transmission from a viremic animal (#280 or #284) as animals were group housed. However, in a previous experiment to assess direct transmission between lambs no direct transmission was observed (Wichgers Schreur et al., 2016). Estimates from these experiments, in which two replicates of seven and eight animals were exposed to four infectious individuals, indicate that the reproduction number of the direct transmission route is significantly lower than 1 (*p*=0.018, R=0, 95% CI: 0-0.70) (Ball, 1986). Furthermore, hygienic and sterile practices were used during animal care and sampling procedures. The low-level exposure with slightly later onset of viremia in animal 281, and the apparently slow declining viremia in animal 284, raise questions on the virus-host arms race and the resulting heterogeneity in the viremia trajectory from virus – host interactions especially when exposure levels are low (Wang et al., 2022). The broad range of vector species involved with RVFV transmission, also implies that animals may well be exposed to mosquitoes with varying levels of competence (Turell et al., 2008; Drouin et al., 2022). In general, gaining understanding of the range of infection outcomes that may arise from mosquito-borne RVFV infection and how this is coupled to heterogeneity within mosquitoes (i.e., viral dose) can help decipher the transmission landscape of this virus.

We estimated an upper boundary for the host-to-mosquito transmission efficiency (*c*) by estimating this parameter at peak viremia of needle-inoculated sheep (31%, 95%CI: 22 - 42%). Peak viremia most likely represents the most contagious period. A host’s transmission potential over their full infectious period (c*r^-1^, net infectiousness) will be lower than expected based on this estimate of *c*. Estimates of net infectiousness derive from the full trajectory of increase and decline of infectious viral particles over the course of the infectious period. The viremia levels are coupled with estimations of how these levels relate to transmission efficiency. This dose response relationship has been estimated for RVFV based on a meta-analysis of available literature and presents a composite estimate across vertebrate species and the *Aedes* genera (Cecilia et al., 2022). Our results (31% from 4.35 to 7.15 log_10_ TCID_50_/ml) are consistent with the described relationships. Estimates of *c* across the full infectious period would aid in getting a better assessment of the net infectiousness and associated dose response relationships. In this study only one full viral trajectory was observed for the low exposure group. The absence of additional complete viral trajectories limits the ability to estimate net infectiousness (using published dose response relationships) and assess how mosquito exposure affects transmission potential.

Using the transmission efficiency estimates from our host-mosquito-host model system in a RossMcDonald-type R_0_ calculation, illustrated that RVFV (vectored by a mosquito with similar transmission potential as *Ae. aegypti*) could invade naive areas if favorable conditions are met. These conditions include temperatures conducive to efficient transmission, sufficiently high mosquito-to-host ratios, and large proportions of mosquito bites on competent hosts. The local vulnerability to sustained pathogen transmission is particularly sensitive to the vector-to-host ratio. This is because a sheep needs to be bitten twice to fulfil the transmission cycle: once to become infected and once to contribute to onward transmission of the pathogen. In that transmission chain the number of bites received by a sheep is proportional to the vector-to-host ratio (i.e, while a mosquito will not feed more often when more hosts are available, the chance for an individual sheep to be fed on is higher if there are sizably more mosquitoes than sheep). All these conditions are context specific. For example, most mosquitoes have a host preference, but many will divert from this preferred species depending on local host availability. This was illustrated in (Tandon and Ray, 2000), where 82% of highly anthropophilic *Ae. aegypti* caught in a cattle-shed had fed on cattle. Furthermore, here we used a theoretical model whereby bites were homogeneously distributed over a host population of one species, i.e., every individual receives the same number of bites. In nature bites are often heterogeneous distributed, with some individuals receiving many bites and others few, affecting pathogen invasion and transmission at population levels (Woolhouse et al., 1997). Together this reinforces that field observations are essential for translation of experimental data to epidemiological parameters and outcomes.

With this host-mosquito-host transmission model we aimed to reproduce the RVFV transmission cycle in a way that best mimics natural virus exposure within our logistic boundaries. Mirroring natural transmission cycles in studies create challenging logistics; host-mosquito-host studies require dedicated facilities, a relatively long timeline, two carefully timed mosquito feeding events, the survival of mosquitoes to feed a second time, among other challenges. To enhance feasibility, deviations from natural systems must be made in model systems. For example, the sheep used in our study are native to the Netherlands and not common in RVFV endemic countries. Further, although this lab-reared *Ae. aegypti* strain can transmit RVFV, other *Aedes* species are more important for RVFV transmission in endemic areas. We chose *Ae. aegypti* as our model species, because this species is easy to maintain in laboratory settings, willing to feed at least twice in captivity and capable of transmitting RVFV to ruminant species (Wichgers Schreur et al., 2021, and this paper). Replacing host and vector species with the species of interest could improve the relevance of the transmission estimates. However, with a relative promiscuous virus, like RVFV, it is impossible to test all possible, and even all relevant, combinations. Moreover, many mosquito species are not suitable for lab rearing and/or are not capable of surviving in laboratory settings to live past the extrinsic incubation period. Furthermore, we tested at 28˚C, higher and lower temperatures likely affect transmission efficiency estimates as well (Tesla et al., 2018). This temperature – transmission relationship could be further explored in future studies. Furthermore, the extended observation period in the low exposure group highlighted that, despite animal welfare and cost concern, prolonged observations may be warranted to address questions on the progression of infection after low exposure. Specifically, the relationship between exposure dose, viremia (including how viremia levels decline) and onward transmission can be extrapolated more precisely. Thus, although model systems cannot capture reality, they are useful to explore coupled heterogeneities (exposure, viremia, transmission) and establish parameter estimates that can be used in epidemiological models.

Mathematical model parameters are often informed by experiments not designed for this purpose. Consequently, estimates may misrepresent the efficiency of transmission or outcomes of infections, for instance due to the use of high inoculation dosages or artificial transmission routes. In this experimental sheep-mosquito-sheep design, we have successfully reproduced RVFV infection in sheep by the bite of a single mosquito, the most likely modality of RVFV infection in ruminants. This design could be used for future research on different host and vector combinations and expanded to examine the drivers behind the heterogeneous outcomes, such as observed in the low exposure group. Individual-level transmission parameters, such as described here, should always be put in the ecological context where the virus is present or at risk of emerging. Teams of epidemiologists, entomologists, ecologists, and virologists, among others, can help to synthesize knowledge across disciplines and put these in an epidemiological perspective meaningful for animal and public health decision makers.

## Data availability statement

The datasets and R code for assessing the relationships between mosquito bites and infection are available at Open Science Framework https://osf.io/8vuax/.

## Ethics statement

The animal study was approved by the Animal Ethics Committees of Wageningen Research. The animal experiment was conducted in accordance with European regulations (EU directive 2010/63/EU) and the Dutch Law on Animal Experiments (WoD, ID number BWBR0003081). Permissions were granted by the Dutch Central Authority for Scientific Procedures on Animals (Permit Numbers: AVD4010020185564 and AVD4010020187168). The study was conducted in accordance with the local legislation and institutional requirements.

## Author contributions

Conceptualization: QB, JK, MJ, CK, LK, and PW; Data Curation: PW, QB, and GB; Formal Analysis: QB and GB; Funding Acquisition: JK, QB, GB, and MJ; Investigation: JK, PW, LK, and RV; Visualization: QB and GB; Writing – Original Draft preparation: QB, GB, and PW; Writing – Review and Editing: JK, CK, LK, RV, and MJ. All authors contributed to the article and approved the submitted version.
