# Supplementary material for: Quantifying Rift Valley fever virus transmission efficiency in a lamb-mosquito-lamb model
Source: Front Cell Infect Microbiol. 2023 Dec 18;13:1206089. doi: 10.3389/fcimb.2023.1206089 (PMC10759236; doi:10.3389/fcimb.2023.1206089)
Supplement: Supplementary file 1 [file DataSheet_1.pdf]

Supplemental files

Supplemental files 1
Supplemental Table 1: Estimate of *b* under different mosquito infection criteria 2
Supplemental Table 2: Donor sheep Rift Valley fever virus titer 3
Supplemental Table 3: Pre-challenge mosquito infection check 4
Supplemental Table 4: Summary of infectious bites and outcome 5
Supplemental Figure 1 6

Supplemental Table 1: Estimate of *b* under different mosquito infection criteria

Bold values are reported in the manuscript.

Hypotheses i and ii were assessed for different mosquito infection criteria. The number of infectious bites per individual in the low exposure group was set to 1, or the number of mosquitoes with PCR positive bodies was used. The number of infectious bites per individual in the high exposure group was a multiplication of 1) the number of mosquitoes a sheep was exposed to, or the number of mosquitoes that had taken a blood meal (engorged), and 2) the proportion of mosquitoes with RVFV particles in saliva or the proportion of mosquitoes with PCR positive bodies. The estimated proportion (mean), lower and upper confidence interval values were used to capture conservative and optimistic scenarios.

Hypothesis	*b*	CI low	CI high	AIC	Low_exposure	High_Exposure_type	High_Exposure_proportion	High_Exposure_estimate
H2	0.29	0.14	0.54	15.82	Set to 1	Exposed	PCR_body	Mean
H2	0.29	0.14	0.54	15.86	Set to 1	Exposed	TCID50_saliva	UpperCI
H2	0.29	0.14	0.54	15.92	Set to 1	Engorged	PCR_body	Mean
H2	0.30	0.15	0.55	16.02	Set to 1	Engorged	TCID50_saliva	UpperCI
H2	0.32	0.16	0.56	16.33	Set to 1	Exposed	TCID50_saliva	Mean
H2	0.18	0.08	0.33	16.35	Set to 1	Exposed	PCR_body	UpperCI
H2	0.33	0.17	0.56	16.54	Set to 1	Engorged	TCID50_saliva	Mean
H2	0.18	0.09	0.34	16.55	Set to 1	Engorged	PCR_body	UpperCI
H2	0.33	0.17	0.56	16.58	Set to 1	Exposed	PCR_body	LowerCI
H2	0.34	0.18	0.57	16.79	Set to 1	Engorged	PCR_body	LowerCI
H2	0.36	0.20	0.59	17.39	Set to 1	Exposed	TCID50_saliva	LowerCI
H2	0.37	0.20	0.60	17.68	Set to 1	Engorged	TCID50_saliva	LowerCI
H2	0.22	0.10	0.43	17.75	PCR positive bodies	Exposed	PCR_body	UpperCI
H2	0.22	0.10	0.43	17.86	PCR positive bodies	Engorged	PCR_body	UpperCI
H2	0.24	0.12	0.44	18.25	PCR positive bodies	Exposed	PCR_body	Mean
H2	0.24	0.12	0.44	18.32	PCR positive bodies	Exposed	TCID50_saliva	UpperCI
H2	0.25	0.12	0.45	18.42	PCR positive bodies	Engorged	PCR_body	Mean
H2	0.25	0.13	0.45	18.60	PCR positive bodies	Engorged	TCID50_saliva	UpperCI
H2	0.27	0.14	0.47	19.10	PCR positive bodies	Exposed	TCID50_saliva	Mean
**H2**	**0.28**	**0.15**	**0.47**	**19.42**	**PCR positive bodies**	**Engorged**	**TCID50_saliva**	**Mean**
H2	0.28	0.15	0.48	19.48	PCR positive bodies	Exposed	PCR_body	LowerCI
H2	0.29	0.15	0.48	19.79	PCR positive bodies	Engorged	PCR_body	LowerCI
H2	0.31	0.17	0.50	20.66	PCR positive bodies	Exposed	TCID50_saliva	LowerCI
H2	0.32	0.17	0.51	21.06	PCR positive bodies	Engorged	TCID50_saliva	LowerCI
**H1**	**0.57**	**0.36**	**0.77**	**30.68**				

H1: *P*(inf) independent of *n* the number of infectious mosquito bites; H2: *P*(inf) dependent on *n*; *b* RVFV transmission efficiency from vector to host; CI: confindence interval

Supplemental Table 2: Donor sheep Rift Valley fever virus titer

Individual	Log_{10} copies / ml	Log_{10} $TCID^{50}$/ml	Mosquitoes fed
271	8.07	4.35	288
272	9.13	5.98	102
273	8.70	7.15	162
274	9.77	6.45	41
275	3.63	1.55	48

Supplemental Table 3: Pre-challenge mosquito infection check

To estimate RVFV infection (positive bodies) and dissemination (positive saliva) rates, 30 mosquitoes were randomly selected for RT-qPCR testing on day 7 post feeding (experimental day -5).

Mosquito	Body	Saliva
1	Neg	Neg
2	Neg	Neg
3	Neg	Neg
4	Neg	Neg
5	2.50E+05	Neg
6	1.37E+05	Neg
7	Neg	Neg
8	Neg	Neg
9	Neg	Neg
10	Neg	Neg
11	4.90E+04	Neg
12	Neg	Neg
13	Neg	Neg
14	7.52E+04	Neg
15	Neg	Neg
16	Neg	Neg
17	Neg	Neg
18	Neg	Neg
19	Neg	Neg
20	Neg	Neg
21	1.91E+05	Neg
22	Neg	Neg
23	Neg	Neg
24	2.50E+05	Neg
25	4.89E+04	Neg
26	Neg	Neg
27	7.20E+05	Neg
28	Neg	Neg
29	Neg	Neg
30	1.29E+02	Neg
**Total**	**9/30**	**0/30**

Supplemental Table 4: Summary of infectious bites and outcome

The number of animals that became viremic after a certain number of infectious bites, and the total number of animals per exposure.

**Group**	**Low exposure**			**High exposure**		
**Number of infectious bites**	1	2	3	7	8	9
**Number of viremic / exposed animals**	3 / 9	0 / 2	0 / 1	1 / 1	4 / 4	4 / 4

Supplemental Figure 1

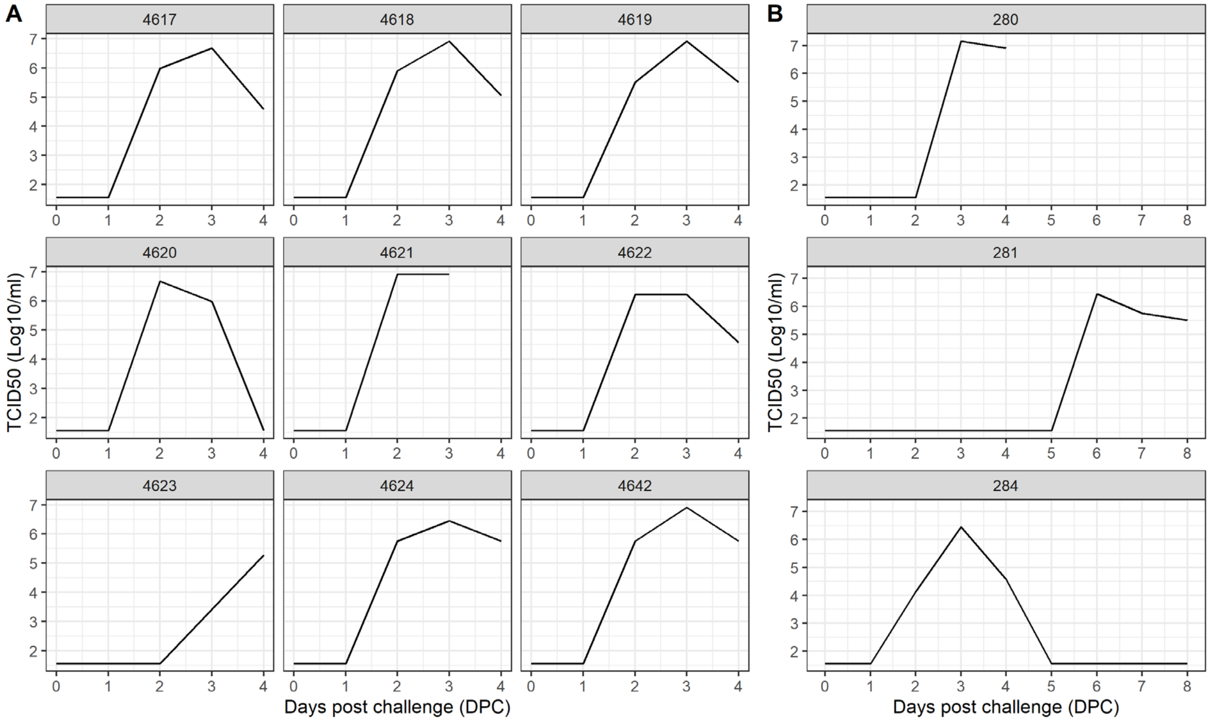

Rift Valley fever virus viremia trajectory of individual sheep in the high exposure (A) and low exposure (B) groups.

Blood samples were taken daily. Animal 4621 and 280 succumbed to infection.
